# Competitive Exclusion Prevents Colonization and Compartmentalization Reduces Transmission of ESBL-Producing *Escherichia coli* in Broilers

**DOI:** 10.3389/fmicb.2020.566619

**Published:** 2020-11-10

**Authors:** Anita Dame-Korevaar, Jannigje G. Kers, Jeanet van der Goot, Francisca C. Velkers, Daniela Ceccarelli, Dik J. Mevius, Arjan Stegeman, Egil A. J. Fischer

**Affiliations:** ^1^Department of Farm Animal Health, Faculty of Veterinary Medicine, Utrecht University, Utrecht, Netherlands; ^2^Department of Bacteriology and Epidemiology, Wageningen Bioveterinary Research, Lelystad, Netherlands; ^3^Laboratory of Microbiology, Wageningen University, Wageningen, Netherlands; ^4^Department of Infectious Diseases and Immunology, Faculty of Veterinary Medicine, Utrecht University, Utrecht, Netherlands

**Keywords:** poultry, compartments, intervention, antimicrobial resistance, ESBL, *Escherichia coli*, colonization

## Abstract

Extended spectrum beta-lactamase (ESBL)-producing bacteria are resistant to extended-spectrum cephalosporins and are common in broilers. Interventions are needed to reduce the prevalence of ESBL-producing bacteria in the broiler production pyramid. This study investigated two different interventions. The effect of a prolonged supply of competitive exclusion (CE) product and compartmentalization on colonization and transmission, after challenge with a low dose of ESBL-producing *Escherichia coli*, in broilers kept under semi-field conditions, were examined. One-day-old broilers (Ross 308) (*n* = 400) were housed in four experimental rooms, subdivided in one seeder (S/C1)-pen and eight contact (C2)-pens. In two rooms, CE product was supplied from day 0 to 7. At day 5, seeder-broilers were inoculated with *E. coli* strain carrying *bla*_*CTX–M–*__1_ on plasmid IncI1 (CTX-M-1-*E. coli*). Presence of CTX-M-1-*E. coli* was determined using cloacal swabs (day 5–21 daily) and cecal samples (day 21). Time until colonization and cecal excretion (log_10_ CFU/g) were analyzed using survival analysis and linear regression. Transmission coefficients within and between pens were estimated using maximum likelihood. The microbiota composition was assessed by 16S ribosomal RNA gene amplicon sequencing in cecal content of broilers on days 5 and 21. None of the CE broilers was CTX-M-1-*E. coli* positive. In contrast, in the untreated rooms 187/200 of the broilers were CTX-M-1-*E. coli* positive at day 21. Broilers in C2-pens were colonized later than seeder-broilers (Time to event Ratio 3.53, 95% CI 3.14 to 3.93). The transmission coefficient between pens was lower than within pens (3.28 × 10^–4^ day^–2^, 95% CI 2.41 × 10^–4^ to 4.32 × 10^–4^ vs. 6.12 × 10^–2^ day^–2^, 95% CI 4.78 × 10^–2^ to 7.64 × 10^–2^). The alpha diversity of the cecal microbiota content was higher in CE broilers than in control broilers at days 5 and 21. The supply of a CE product from day 0 to 7 prevented colonization of CTX-M-1-*E. coli* after challenge at day 5, likely as a result of CE induced effects on the microbiota composition. Furthermore, compartmentalization reduced transmission rate between broilers. Therefore, a combination of compartmentalization and supply of a CE product may be a useful intervention to reduce transmission and prevent colonization of ESBL/pAmpC-producing bacteria in the broiler production pyramid.

## Introduction

Extended spectrum beta-lactamase and plasmid AmpC beta-lactamase (ESBL/pAmpC)-producing bacteria are resistant to extended-spectrum cephalosporins (ESC). ESBL/pAmpC-producing bacteria are present in humans, animals and the environment (Blaak et al., 2015). Poultry is known as a source of ESBL/pAmpC-producing bacteria and high prevalence in poultry and poultry products have been reported in several European countries, as reviewed by Saliu et al. (2017). ESBL/pAmpC-producing bacteria are present at all levels of the broiler production pyramid (Dierikx et al., 2013; Agerso et al., 2014; Nilsson et al., 2014; Zurfluh et al., 2014a, b; Projahn et al., 2018). Different routes of transmission within the broiler production pyramid have been described, for example between generations, via the hatcheries, and on and between farms (Dame-Korevaar et al., 2019a). As a consequence, introduction of ESBL/pAmpC-producing bacteria can occur at several levels of the broiler production pyramid, for example at the farm or at the hatchery. A recent study estimated that, based on the proportional similarity index (PSI), the average transfer of ESBL/pAmpC genes between subsequent generations in the broiler production pyramid is almost 50% (Apostolakos et al., 2019). However, for most of the routes it is unknown to what extent they contribute to the presence of ESBL/pAmpC-producing bacteria in the broiler production pyramid. In Netherlands, antimicrobial resistance in broilers has decreased significantly since 2010 (Hesp et al., 2019), following the trend of reduced antimicrobial usage. However, additional interventions are needed to further reduce this prevalence in the broiler production pyramid.

Interventions can aim to reduce exposure of broilers to ESBL/pAmpC-producing *Escherichia coli*. This can be done by improving biosecurity. For example hygiene barriers can help reduce exposure to bacteria from the farm environment, or by cleaning and disinfection between production rounds. However, even after cleaning and disinfection, ESBL/pAmpC-producing bacteria might remain in the poultry house and result in colonization of the new flock (Daehre et al., 2018). In addition, housing measures may reduce the prevalence of ESBL/pAmpC-producing *E. coli* in poultry flocks. In turkeys, subdividing the flock was associated with a reduced risk for the presence of resistant *E. coli* on the farm (Jones et al., 2013). Experimental studies showed that spatial separation between infectious and susceptible animals reduced the transmission rate of *Campylobacter* in broilers (van Bunnik et al., 2012) and *Streptococcus suis* in pigs (Dekker et al., 2013). Further, interventions aiming at preventing colonization by ESBL/pAmpC-producing *E. coli* in broilers have been described, such as acid-based feed additives (Roth et al., 2017) or competitive exclusion (CE) products (Nuotio et al., 2013; Ceccarelli et al., 2017; Methner et al., 2019; Dame-Korevaar et al., 2020). CE products are aimed at establishing a natural community of intestinal bacteria to protect broilers from colonization by invaders (Nurmi et al., 1992). In modern broiler production, due to strict hygiene practices in commercial hatcheries, the initial bacterial load to colonize the chicken intestinal tract shortly after hatch is low (Varmuzova et al., 2016; Donaldson et al., 2017). Eggs are usually disinfected to remove bacterial contamination before placement in the hatcher. Consequently, the chicks are exposed mostly to bacteria from environmental sources rather than parental sources upon hatching. Microbial treatment supplied after hatch has been shown to affect the development of bacterial taxa found in growing chickens (Ballou et al., 2016; Schokker et al., 2017). This suggests that early supply of CE products might influence microbiota composition and act as a possible intervention to prevent colonization by ESBL/pAmpC-producing *E. coli* in young broilers. A single supply of CE product before challenge with a high dose of ESBL-producing *E. coli* has already showed to reduce colonization, cecal and fecal excretion (CFU/g), as well as transmission of ESBL-producing *E. coli* (Nuotio et al., 2013; Ceccarelli et al., 2017; Methner et al., 2019). Additionally, CE products resulted in a reduced intestinal and cecal excretion (CFU/g) after challenge with pathogenic *E. coli* (Hofacre et al., 2002). A prolonged supply of CE product via the drinking water to broilers kept in isolators, from day of hatch until day 14 resulted in a delay and even prevention of colonization after challenge with a in the field realistic low dose of ESBL-producing *E. coli* (Dame-Korevaar et al., 2020).

The aim of this study was to determine the effect of interventions on colonization and transmission of ESBL-producing *E. coli* in young broiler chicks kept under semi-field circumstances. Two interventions were included: (1) prolonged supply of CE product from day of hatch until day 7, and (2) compartmentalization of a broiler flock. To investigate the effect of CE product on microbial composition, microbiota in cecal content was assessed before and after challenge by 16S ribosomal RNA (rRNA) gene amplicon sequencing.

## Materials and Methods

### Ethics of Experimentation

Broilers were observed daily and the presence of clinical signs, abnormal behavior and mortality was recorded. The study protocol was approved by the Dutch Central Authority for Scientific Procedures on Animals and the Animal Experiments Committee of Utrecht University (Utrecht, Netherlands) under registration number AVD108002015314; all procedures were done in full compliance with Dutch legislation, and is thus compliant with legislation in the EU directive 2016/3/EU.

### Birds, Housing and Management

**FIGURE 1:**
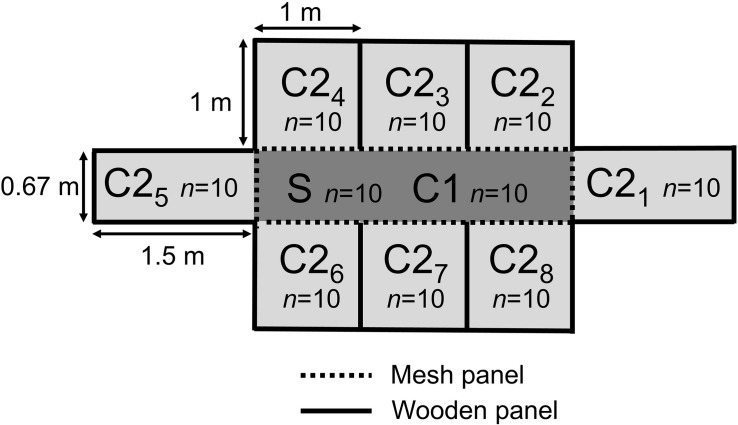
Schematic representation of the experimental set up of one of four broiler rooms (1–4). Each room was subdivided in nine pens, with one seeder (S/C1) pen in the middle (2 m^2^) (*n* = 10 S-broilers, 10 C1-broilers), surrounded by eight contact (C2) pens (1 m^2^) (*n* = 10 broilers per pen). The S/C1-pen was separated from the C2-pens by 80 cm high mesh panels. The C2-pens were separated from each other by 80 cm high wooden panels.

Conventional broiler chicks (Ross 308, *n* = 416), from a parent stock flock of 37 weeks of age, were transported directly after hatch to the animal facilities (Utrecht University, Utrecht, Netherlands). Upon arrival, the broilers were individually tagged, weighed, and randomly divided over four experimental rooms (*n* = 104 broilers per room). Each room was subdivided into nine pens, with one seeder (S/C1)-pen in the middle (2 m^2^, *n* = 24 broilers), surrounded by eight contact (C2)-pens (1 m^2^, *n* = 10 broilers per pen) (Figure 1). The S/C1-pen was separated from the C2-pens by a mesh panel (30 cm solid panel at the bottom, 40 cm mesh panel, 10 cm solid panel on top). Feed and water systems were also separated, and strict hygiene measures between pens were taken. No direct contact between the broilers was possible, but small particles (e.g., litter, dust) could be transferred between pens potentially. The C2-pens were separated from each other with wooden panels of 80 cm height, assuming no contact and no spread of particles was possible. At day 5, just before challenge with ESBL-producing *E. coli*, the number of broilers in the S/C1-pen was reduced to 20, by removing the surplus broilers. Ten of the remaining 20 broilers in each S/C1-pen were randomly selected and transported to four separate isolators. In these isolators, the broilers (seeder (S) broilers) were inoculated with CTX-M-1-*E. coli* and after 1 h moved back to the original S/C1-pens (see section “*E. coli* Challenge”). Before the start of the experiment the parent flock, hatchery and research facilities were tested for the absence of ESBL/pAmpC-producing bacteria.

Broilers were housed on fine wood shavings. A standard broiler diet without any antibiotics or coccidiostats, radiated with 9 Gy, was available *ad libitum*. The intervention was supplied in the drinking water (see section “Intervention Competitive Exclusion”); therefore, drinking water was not available *ad libitum* during the first seven days of the experiment in both intervention and control groups. Five out of 400 broilers died or were euthanized before the end of the experiment due to causes unrelated to the experiment, but by common causes in young broilers, i.e., not starting to eat or drink or omphalitis.

### Intervention Competitive Exclusion

In two of four rooms a CE product was supplied, containing “natural, live intestinal microflora derived from specific pathogen free (SPF) chickens and manufactured by fermentation” (Aviguard^®^, MSD Animal Health, Netherlands). From the moment of arrival in the rooms (day 0, 10:00 a.m.) until day 7, (4:00 p.m.), CE product was supplied in the drinking water, twice per day. Water solutions containing the CE product were prepared in pre-dilution, with a dose level according to recommendations of the manufacturer, i.e., 0.125 g CE product per 10 broilers. The amount of drinking water was restricted between day 0 and 7, based on the expected water consumption of 10 (C2-pen) and 20 (S/C1-pen) broilers in a pen to ensure that all supplied CE product would be consumed.

### *E. coli* Challenge

Broilers were challenged with *E. coli* strain E38.27, which carries the ESBL gene *bla*_*CTX–M–*__1_ on an IncI1 plasmid (CTX-M-1-*E. coli*), isolated from conventional healthy broilers at slaughter age and resistant to cefotaxime (Dierikx et al., 2010). Oral inoculation of seeder (S) birds was performed on day 5 at 8:00 a.m. using a 1 mL syringe without a needle with 0.5 mL of 10^2^ CFU/mL. The bacterial dilution was measured with the McFarland reader and retrospective colony counting. From 1 h after inoculation onward, 10 contact (C1) birds were exposed to 10 seeder birds, by moving the inoculated seeder birds to the corresponding S/C1-pens containing the contact birds.

### Cloacal and Cecal Samples

Samples were taken using sterile dry cotton swabs (Copan 155C, Copan Diagnostics, United States). Broilers were sampled at day 5 at 4:00 a.m., just before inoculation to confirm absence of ESBL/pAmpC-producing bacteria, and from day 6 until day 21 daily at 8:00 a.m. At day 21, after the last sampling, *post mortem* examination was done within 30 min after euthanasia for each broiler. Broilers were weighed and sex was determined, exterior and interior abnormalities were assessed, and ceca were collected and stored on dry ice for further analysis.

### Microbiota Sample Collection and Analysis

Cecal content samples were collected from five surplus broilers of the control group and from five surplus broilers of the CE intervention group (*n* = 10) at day 5. At day 21, cecal content of all broilers in the S/C1-pen in all four rooms (*n* = 80) was collected. The closed side of one of the two ceca was cut and cecal content was gently squeezed into a 2 mL sterile cryotube and snap frozen on dry ice and stored at −80°C for genomic DNA extraction. To determine the microbial composition of the CE product, Aviguard^®^ was suspended in PBS according to the manufacturer’s instructions and four aliquots of 2 mL were stored at −80°C for bacterial genomic DNA extraction. The full protocol for DNA extraction and determining microbiota composition was previously described (Kers et al., 2019). Briefly, DNA was extracted from 0.25 g cecal content or frozen CE product, using 700 μL of Stool Transport and Recovery (STAR) buffer (Roche Diagnostics Nederland BV, Netherlands). All 94 samples were transferred to a sterile screw-capped 2 mL tube (BIOplastics BV, Netherlands), used for bead beating. The DNA concentrations were measured with a NanoDrop ND-1000 spectrophotometer (NanoDrop^®^ Technologies, United States), and the DNA samples were stored at −20°C until further use. Barcoded amplicons covering the variable regions V5–V6 and primers 784F and 1064R were used for 16S rRNA gene-based microbial composition profiling as previously described (Ramiro-Garcia et al., 2016).

To ensure high quality sequencing data, synthetic communities of known composition were used as positive controls (Ramiro-Garcia et al., 2016) and nuclease free water as negative controls. Sequencing of resulting libraries was performed on Illumina Hiseq 2500 (Eurofins Genomics Germany GmbH). The 16S rRNA data was analyzed using NG-tax 2.0 (Ramiro-Garcia et al., 2016). In short, to generate amplicon sequence variants (ASVs), NG-Tax 2.0 employed a fast *de novo* ASV-picking algorithm. To assign taxonomy the SILVA 128 16S rRNA gene reference database was used (Quast et al., 2013). Bacterial names were identified on the genus-level, however, if this level was unknown, we used the lowest known taxonomic classification. Raw sequence data were deposited into the Sequence Read Archive (SRA) at the NCBI, under accession number PRJNA647260.

### ESBL-Producing *E. coli* Detection

All cloacal samples were enriched in 3 mL Luria-Bertani (LB) broth. After overnight incubation at 37°C, 10 μL broth were inoculated on MacConkey plates supplemented with 1 mg/L cefotaxime and incubated overnight at 37°C. *E. coli* colonies growing on MacConkey plates supplemented with cefotaxime were referred to as CTX-M-1-*E. coli*. If visual assessment was not conclusive on the presence of *E. coli*, colonies were selected for further analyses using MALDI-TOF MS (Bruker Daltonik, Germany).

### ESBL-Producing *E. coli* and Total *E. coli* Quantification

At day 21, content from one of two ceca of 80 selected broilers from rooms 1 and 2 was collected. For both rooms, selection included all broilers (*n* = 20) from the S/C1-pen and additionally 20 broilers from the C2-pens which were excreting CTX-M-1-*E. coli*. Samples were processed as previously described (Dame-Korevaar et al., 2019b). Concentrations of ESBL-producing *E. coli* and total *E. coli* were determined semi-quantitatively. CFU/gram feces was calculated based on the highest dilution showing growth of typical *E. coli* colonies (Jett et al., 1997) and the weight of the feces on the swabs or the amount of cecal content collected (Ceccarelli et al., 2017). *E. coli* colonies growing on MacConkey plates supplemented with cefotaxime were referred to as CTX-M-1-*E. coli*. If visual assessment was not conclusive on the presence of *E. coli*, colonies were selected for further analyses using MALDI-TOF MS.

### Statistical Analysis

Statistical analyses were performed in R, version 3.4.3 (RStudio Team, 2016), using packages survival, phyloseq, microbiome, and vegan. Regressions were performed with function glm().

#### Time Until Colonization

Time until colonization was analyzed using parametric survival regression with an accelerated failure time model using a Weibull distribution (Kalbfleisch and Prentice, 2002). The hazard ratio was expected to be non-proportional during the experiment, because of the compartmentalization. This accelerated failure time model models the effect of the variables on the acceleration or deceleration of the time until colonization with CTX-M-1-*E. coli*. Colonization of individual broilers was measured as excretion of CTX-M-1-*E. coli* and time until colonization was defined as the time point of the first cloacal swab of two consecutive cloacal swabs tested positive for CTX-M-1-*E. coli*. If the last swab (day 21) and the ceca tested positive, broilers were assumed to be colonized at day 21. If only the ceca tested positive, broilers were not included as colonized birds within the time span of the experiment.

#### Microbiota Composition

Differences of relative abundance were tested with Wilcoxon rank-sum test and corrected for multiple testing using Benjamini–Hochberg (BH) procedure. Alpha and beta diversity metrics were calculated and univariate and multivariate statistical analyses were applied to determine differences in the cecal microbiota. Alpha diversity (within sample richness) was determined using Faiths phylogenetic diversity, taking into account the phylogenetic relatedness (Faith, 2007). Differences in alpha diversity were tested using a non-parametric Kruskal–Wallis test. Beta diversity (between sample differences) was determined using weighted and unweighted UniFrac metrics (Lozupone et al., 2007). Principal coordinates analysis (PCoA) was used to visualize the data. To test differences within multivariate community data, non-parametric permutational analysis of variance (PERMANOVA) were used (Anderson, 2001).

#### Transmission Coefficient

The transmission coefficients for within and between pen transmission (β_*within*_ and β_*between*_) were estimated based on a stochastic multi-pen SI model (Klinkenberg et al., 2002; Velthuis et al., 2007) in which the number of new cases is determined by transmission from excreting (I) birds to susceptible (S) birds for a total population of (N) birds, using maximum likelihood estimation.

The probability (*p*_*k*_) for a susceptible animal in pen *k* to become colonized during time interval Δ*t* was calculated based on the force of infection (*foi*) within the pen and between pens (S/C1-pen to C2-pen):

Equation1 $$p_{k}=1-e^{-\left(foi_{\text{within}}+foi_{\text{between}}\right)\text{Δ}t}$$

Two models were used in which the *foi* was based in model 1 on direct transmission or in model 2 on indirect transmission with a build-up of infectivity in the environment. In model 1 *foi* was determined by the proportion of excreting birds in the same pen ($\frac{I_{k}}{N_{k}}$) and the proportions of excreting birds in the adjacent pen connected through a mesh panel ($\frac{I_{\text{adj}}}{N_{\text{adj}}}$) during a time interval Δ*t*:

Equation2 $$p_{k}=1-e^{-\left(\beta_{\text{within}}\frac{I_{k}}{N_{k}}+\beta_{\text{between}}\frac{I_{\text{adj}}}{N_{\text{adj}}}\right)\text{Δ}t}$$

The unit of β_within_ and β_between_ in model 1 is 1/day, and is interpreted as the number of new colonized broilers per day, due to one positive broiler.

In model 2 the *foi* in pen *k* was assumed to be a result of a build-up of infectivity in the environment. The cumulative sum of hours that all excreting birds were excreting in a pen (cumexcrhours_k_) and in the adjacent pen connected with a mesh panel (cumexcrhours_adj_) up to the beginning of the interval was used as a measure for environmental accumulation:

Equation3 $$p_{k}=1-e^{-\left(\beta_{\text{within}}{\text{cumexcrhours}}_{\text{k}}+\beta_{\text{between}}{\text{cumexcrhours}}_{\text{adj}}\right)\text{Δ}t}$$

The unit of β_within_ and β_between_ in model 2 is 1/day^2^ and is interpreted as the number of new colonized broilers per day, caused by each day that one positive broiler has been excreting CTX-M-1-*E. coli* (Dekker et al., 2013; Dame-Korevaar et al., 2020).

#### Cecal Excretion Levels

The differences in cecal content of total *E. coli* and CTX-M-1-*E. coli* (CFU/g) were tested using a linear regression model including the variables room, pen, sex, weight at day of hatch, weight at day 21, type of bird (S, C1, C2) and time until colonization. The best fitting model was obtained by backward selection based on lowest AIC value. The correlation between cecal content of CTX-M-1-*E. coli* and time until colonization was tested using Pearson’s correlation coefficient.

## Results

### Time Until Colonization

**FIGURE 2:**
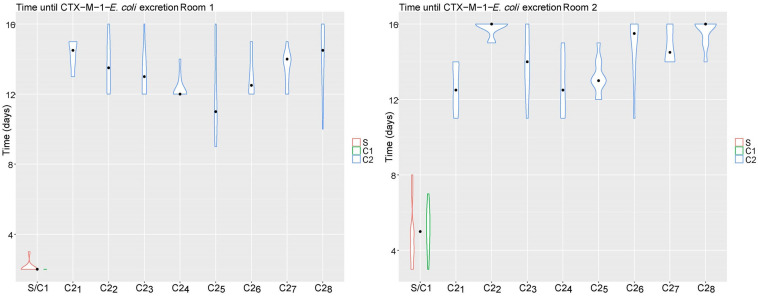
Time until colonization (days) of CTX-M-1-*E. coli* per pen (S/C1, C2_1_, C2_2_, C2_3_, C2_4_, C2_5_, C2_6_, C2_7_, C2_8_) and type of bird [seeder (S), contact 1 (C1), contact 2 (C2)] for room 1 (left) and room 2 (right). The violin plot indicates the total range of observations; the black dot indicates the median.

Broilers in the CE groups (room 3 and 4) were not colonized with CTX-M-1-*E. coli*. In the control groups all broilers in room 1 (*n* = 100), and 87/100 broilers in room 2 were colonized at the end of the experiment (Figure 2). Time until colonization was delayed for broilers in room 2 compared to broilers in room 1 (Time Ratio (TR) 3.00, 95% CI 1.82 to 4.95), and for C2 broilers compared to seeder broilers (TR 3.53, 95% CI 3.14 to 3.93). No difference in time until colonization was observed between seeder and C1 broilers (TR 1.14, 95% CI 1.00 to 1.30). Weight at day of hatch, weight at day 21 and sex did not influence time until colonization (Table 1).

**TABLE 1:** Regression coefficients of time until colonization (95% CI) of CTX-M-1-*E. coli* for an accelerated failure time model.

Variable		Accelerated failure time (days, 95% CI)*
Baseline survival (Room 1, Seeder, Male)		3.00 (1.82–4.95)
Room 2		1.24 (1.15–1.33)
Animal type	Contact 1	1.14 (1.00–1.30)
	Contact 2	3.53 (3.14–3.93)
Sex	Female	0.97 (0.91–1.03)
Bodyweight at day 0		1.00 (0.99–1.02)
Bodyweight at day 21		1.00 (1.00–1.00)

*Accelerated failure time, indicating the acceleration or deceleration of the time until colonization with CTX-M-1-E. coli in the mentioned group compared to the reference (baseline) group. A ratio of >1 indicates a longer time until colonization, and a ratio of <1 indicates a shorter time until colonization.

### Microbiota Composition in Cecal Content

**FIGURE 3:**
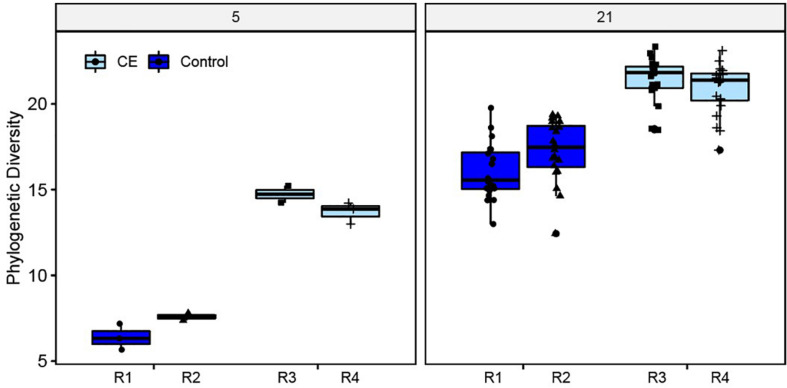
Alpha (phylogenetic) diversity of cecal microbiota at day 5 (*n* = 5 broilers per intervention) and day 21 (*n* = 40 broilers per intervention), for the control (rooms (R) 1, 2) and intervention groups (rooms (R) 3, 4).

The alpha diversity (phylogenetic diversity) was higher in cecal content samples of the broilers supplied with CE product (CE broilers) compared to the control broilers on day 5 and day 21 (Figure 3). On day 21 no differences in alpha diversity between the two intervention rooms were observed (*X*^2^ = 1.90, *p* = 0.17), but the control broilers in room 1 had a lower alpha diversity than control broilers from room 2 (*X*^2^ = 4.92, *p* = 0.03). Within rooms, no differences between seeder and contact (C1) broilers were found. Weight at day of hatch, weight at day 21 and sex did not influence the alpha diversity (data not shown).

**FIGURE 4:**
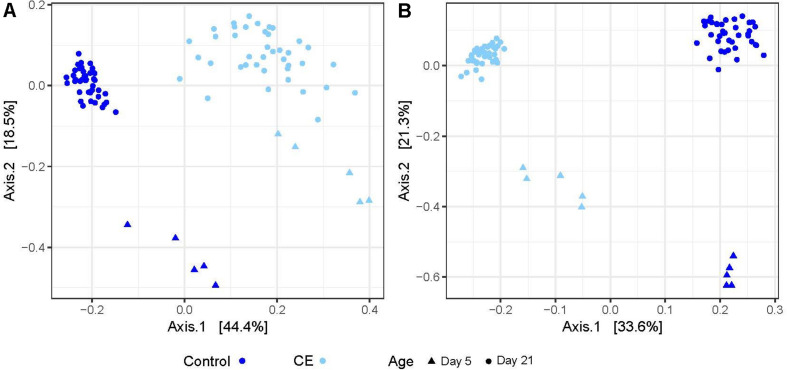
Principal coordinate analysis (PCoA) of microbiota composition based on weighted UniFrac **(A)** and unweighted UniFrac **(B)** distances between control (dark blue) and CE (light blue) groups. Different symbols indicate different sampling days: triangles are samples of day 5, and circles are samples of day 21.

In weighted and unweighted UniFrac (wuf and uf) distance based analysis, the supply of the CE product explained 60% (wuf) and 69% (uf) of the variation between the cecal content samples on day 5 (Figure 4, principal coordinate analysis (PCoA), PERMANOVA, wuf: *R*^2^ = 0.598, *p* = 0.009, uf: *R*^2^ = 0.688, *p* = 0.008). On day 21, application of the CE product explained 46% (wuf) and 51% (uf) of the variation between the cecal content samples (Figure 4, PERMANOVA, uf:*R*^2^ = 0.461, *p* = 1.0 × 10^–4^, wuf: *R*^2^ = 0.510, *p* = 1.0 × 10^–4^). Within rooms, being a seeder or contact broiler did not explain any of the variation between the cecal content samples. The variation between the two control groups was larger than between the two intervention rooms (wuf: *R*^2^ = 0.351 vs. *R*^2^ = 0.210).

**FIGURE 5:**
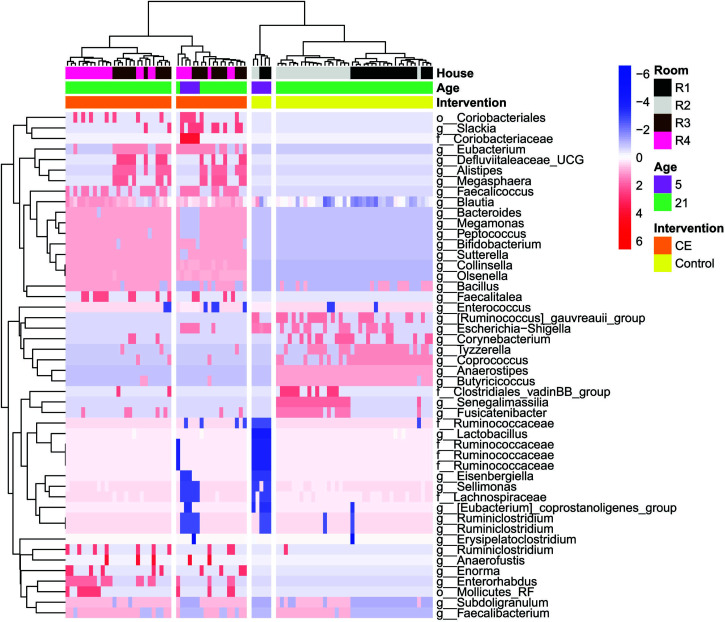
Heatmap representing the abundance of amplicon sequence variants (ASVs) in all individual broiler chickens analyzed (*n* = 90). Only ASVs that are significantly different at day 5 and day 21 between CE and control are shown (Wilcoxon rank-sum test, adjusted *p*-values are corrected *p*-values for multiple testing, Benjamini–Hochberg, *p* < 0.05). Each red, white, or blue rectangle represents the relative abundance of a genus in an individual broiler. Clustering of broilers is based on Ward’s minimum variance method and based on weighted UniFrac distances matrix.

The heatmap (Figure 5) shows all genera that significantly differed in relative abundance between CE broilers and control broilers at day 5 and 21. Selection of the first four clusters reveal two clusters with control broilers: one for the broilers of 5 days of age, and one for the broilers of 21 days of age. The other two clusters consist of CE broilers, one cluster contains broilers of both 5 and 21 days of age, while the second cluster contains only CE broilers of 21 days of age.

In the CE product, 22 different genera were identified (Table 2). Of these genera, five were more abundant in CE broilers than in control broilers at day 5: *Collinsella*, *Eubacterium*, *Flavonifractor*, *Lachnoclostridium*, and *Lactobacillus*. At day 21, genera *Eubacterium coprostanoligenes*, *Bacteroides*, *Collinsella*, *Enterococcus*, *Eubacterium*, *Megamonas*, *Megasphaera*, *Slackia*, and *Sutterella* were more abundant in CE than in control broilers (Table 2).

**TABLE 2:** Relative abundance and standard deviation (SD) of genera that were present in the CE product, and the significantly different relative abundance in cecal content of CE broilers vs. control broilers at day 5 (*n* = 10) and 21 (*n* = 80).

Relative abundance CE product			Change in relative abundance CE vs. control broilers					
			Day 5 Relative abundance (%)			Day 21 Relative abundance (%)		
		
Genera	Relative abundance (%)	SD (%)	*Control*	*CE*	*p-value*	*Control*	*CE*	*p-value*
*[Eubacterium] coprostanoligenes group*	0.65	0.22				0.70	1.11	8.05 × 10^–07^
*Bacteroides*	0.47	0.06				–	1.12	9.06 × 10^–15^
*Blautia*	0.30	0.09				18.48	6.64	2.75 × 10^–12^
*Candidatus_Soleaferrea*	0.39	0.06						
*Clostridium sensu stricto 1*	2.77	0.45	14.67	0.72	0.03	0.04	–	0.04
*Clostridium sensu stricto 2*	0.72	0.12						
*Collinsella*	0.53	0.07	–	12.98	0.03	–	4.28	3.34 × 10^–15^
*Enterococcus*	10.80	1.07	31.76	13.40	0.03	0.46	0.94	2.67 × 10^–03^
*Erysipelatoclostridium*	2.53	0.09			0.03	1.84	0.99	0.03
*Escherichia-Shigella*	0.57	0.02	15.09	0.99	0.03	0.09	3.72 × 10^–03^	2.04 × 10^–04^
*Eubacterium*	0.66	0.04	–	3.31	0.03	–	0.20	2.22 × 10^–07^
*Flavonifractor*	1.02	0.14	–	0.79	0.03			
*Lachnoclostridium*	9.78	0.93	–	1.77	0.03			
*Lactobacillus*	14.96	1.33	–	10.77	0.03			
*Megamonas*	1.55	0.56				–	10.36	3.34 × 10^–15^
*Megasphaera*	3.30	0.74				–	0.27	3.82 × 10^–05^
*Negativicoccus*	3.62	0.66						
*Oscillibacter*	1.94	0.18						
*Peptostreptococcus*	30.97	4.04						
*Sellimonas*	1.31	0.38				1.14	0.64	2.60 × 10^–04^
*Slackia*	0.34	0.09				–	0.03	7.57 × 10^–03^
*Sutterella*	1.76	0.21				–	0.99	3.34 × 10^–15^
uncultured	4.45	3.56						
Unknown	0.08	0.09						

*Results are based on differences of relative abundance tested with Wilcoxon rank-sum test. Adjusted p-values (<0.05) are corrected for multiple testing with Benjamini–Hochberg (BH). – = not detected.*

### Transmission

Broilers in the CE groups (room 3 and 4) were not colonized with CTX-M-1-*E. coli*, and transmission was thus not observed.

In the control groups, the transmission coefficient between pens (β_*between*_) was lower than the transmission coefficient within pens (β_*within*_) for both models. Model 2, with accumulated environmental transmission, was preferred over model 1, assuming direct transmission (AIC 402.1 vs. 438.1, Table 3). For model 2, β_*between*_ was 3.28 × 10^–4^ day^–2^ (95% CI 2.41 × 10^–4^ to 4.32 × 10^–4^) and β_*within*_ was 6.12 × 10^–2^ day^–2^ (95% CI 4.78 × 10^–2^ to 7.64 × 10^–2^) (Table 3).

**TABLE 3:** Transmission coefficients (β_*within*_ and β_*between*_, 95% CI) using an SI-model for transmission based on the proportion of excreting birds (model 1) and the accumulative excretion time (model 2).

Transmission coefficient (β, 95% CI)				

	Unit*	β_*within*_ (95% CI)	β_*between*_ (95% CI)	AIC
Model 1 proportion excreting birds	day^–1^	1.31 (1.07–1.59)	0.03 (0.02–0.04)	438.1
Model 2 accumulative excretion time	day^–2^	6.12 × 10^–2^ (4.78 × 10^–2^–7.64 × 10^–2^)	3.28 × 10^–4^ (2.41 × 10^–4^–4.32 × 10^–4^)	402.1

* The unit in model 1 is 1/day, and is interpreted as the number of new colonized broilers due to one positive broiler per day. The unit in model 2 is 1/day^2^ and is interpreted as the number of new colonized broilers caused by each day that one positive broiler has been excreting.

### Cecal Excretion Levels

Mean CTX-M-1-*E. coli* (log_10_ CFU/g) was lower in cecal samples from broilers from C2-pens than from the S/C1-pen, except for pen C2_6_ and C2_7_ (Table 4). CTX-M-1-*E. coli* (log_10_ CFU/g) was lower in cecal samples from broilers kept in room 2 than broilers kept in room 1 (estimate −0.52, 95% CI −0.91 to −0.13 log_10_ CFU/g). Broilers with a higher bodyweight at day of hatch had slightly higher cecal CTX-M-1-*E. coli* levels (estimate 0.08, 95% CI 0.01 to 0.15 log_10_ CFU/g). Cecal CTX-M-1-*E. coli* levels were correlated with time until colonization, the shorter the time until colonization, the higher the cecal level (*r* = −0.60, 95% CI −0.73 to −0.43). Mean *E. coli* levels in cecal content did not differ between rooms or pens.

**TABLE 4:** Parameter estimates for cecal content levels at day 21 (log_10_ CFU/g cecal content, 95% CI) of CTX-M-1-*E. coli* (*n* = 75) using a linear regression model.

Variable	Estimate CTX-M-1-*E. coli* (95% CI)
Room 1, pen Seeder/C1 (intercept)	3.95 (0.93 to 6.98)
Room 2	−0.52 (−0.91 to−0.13)
Pen C2_1_	−1.17 (−1.91 to −0.43)
Pen C2_2_	−2.28 (−3.23 to −1.33)
Pen C2_3_	−1.85 (−2.95 to −0.75)
Pen C2_4_	−2.01 (−2.64 to −1.39)
Pen C2_5_	−0.86 (−1.40 to −0.33)
Pen C2_6_	−0.59 (−1.52 to 0.33)
Pen C2_7_	0.34 (−0.77 to 1.46)
Pen C2_8_	−2.69 (−3.49 to−1.88)
Bodyweight day of hatch (day 0)	0.08 (0.01 to 0.15)

## Discussion

The supply of CE product via drinking water from day of hatch until day 7 prevented colonization of broilers with ESBL-producing *E. coli* after challenge of seeder birds. In the control group, 93.5% of the broilers were colonized at the end of the experiment. These results are in line with earlier experiments within isolators, in which a continuous supply of CE product during the first 14 days was able to prevent colonization (Dame-Korevaar et al., 2020). In the isolators in which at least one bird was colonized with ESBL-producing *E. coli*, application of CE products reduced the rate of colonization, decreased excretion (CFU/g) and reduced transmission, as previously shown in studies applying a single supply of CE (Hofacre et al., 2002; Nuotio et al., 2013; Ceccarelli et al., 2017; Methner et al., 2019). The enhanced effect of CE product found in this study compared to these earlier studies could have resulted from the prolonged supply, the longer period between start of CE product and exposure to ESBL-producing *E. coli*, or the moment of challenge with ESBL-producing *E. coli* and the low challenge dose used in our study.

The microbiota composition was more diverse in the CE broilers than in the control broilers on day 5 and 21. This supports the hypothesis that microbial diversity plays a role in preventing colonization. Successful CE of ESBL-producing *E. coli* by specific genera being present in the CE broilers could also have prevented colonization. Intestinal colonization with microbiota of adult donor hens is associated with increased resistance against colonization, e.g., with *Salmonella* (Varmuzova et al., 2016). In a study where newly hatched layer chicks were exposed to an adult hen, transfer of microbiota occurred within 24 h of contact and a 1–3 days longer contact period resulted in an even more developed chick microbiota (Kubasova et al., 2019). In our study, supplying a CE product derived from intestinal bacteria of adult chickens possibly increased resistance and prevented colonization with ESBL-producing *E. coli*. The higher diversity observed in broilers at day 5 was maintained during the experiment. At day 21, 2 weeks after the last supply of the CE product, the intestinal microbiota composition was still more diverse in the CE broilers. Next to genera identified in the CE product, also other genera were found to be different between CE and control groups, indicating that the intestinal microbiota of CE broilers was early and persistently different compared to the composition of the microbiota as observed in the control broilers.

Direct competition between specific bacteria and inoculated *E. coli* in CE broilers might have played a role in preventing colonization, including competition for binding sites or limiting nutrients (Nurmi et al., 1992; Callaway et al., 2008). This could be related also with the production of antimicrobial compounds, including volatile fatty acids, inhibiting or eliminating species that compete for the same niche (Callaway et al., 2008). Some genera were present exclusively in the CE product and in CE broilers, but not in control broilers (Table 2). Due to competition, these genera might have prevented colonization. Next to preventing colonization with *E. coli*, CE products have shown to prevent or reduce colonization of different bacteria, e.g., *Salmonella* (Nakamura et al., 2002; Ferreira et al., 2003; Luoma et al., 2017; Markazi et al., 2018) and *Campylobacter* (Schneitz and Hakkinen, 2016).

In the control groups compartmentalization resulted in a significantly lower transmission between pens than within pens. Transmission between pens shows that environmental transmission can occur and presence of ESBL/pAmpC-producing bacteria in litter, air or dust plays a role in transmission (Friese et al., 2013; Laube et al., 2013, 2014; Blaak et al., 2014, 2015; Daehre et al., 2018). Delayed transmission as result of compartmentalization has been described for other pathogens (van Bunnik et al., 2012; Dekker et al., 2013). The effect of compartmentalization can be two-fold: the physical barrier prevents direct contact between broilers, and next to that, during the time needed for transmission between pens to occur, the microbiota of the susceptible broilers might develop further, making it more difficult for ESBL-producing *E. coli* to colonize. In chickens, microbiota in the first week of life contains *Enterobacteriaceae* (Videnska et al., 2014; Ballou et al., 2016; Jurburg et al., 2019) suggesting that *E. coli* can easily colonize during the first week. Older bird might get less susceptible for colonization (Dame-Korevaar et al., 2020), however in our study colonization with ESBL-producing *E. coli* still occurred at 21 days of life, maybe as a result of accumulation of excreted ESBL-producing *E. coli* in the environment. Once transmission between pens occurred, transmission within pens followed rapidly. In room 1, within S/C1-pen transmission occurred very fast: all except one bird were positive at the first sampling after challenge. Therefore, this pen could not be included in the estimation of within pen transmission.

Estimation of the transmission coefficients was done using the proportion of excreting birds (model 1) and the accumulative excretion time (model 2). The model including excretion time fitted better to the observed data, indicating that accumulation of ESBL-producing *E. coli* in the environment most likely plays a role in the transmission within a flock. This increased infectivity by accumulation of the bacteria has been modeled also for other pathogens in pigs and chicken (Lurette et al., 2008; Dekker et al., 2013; van Bunnik et al., 2014). In our model, environmental accumulation is assumed to be unlimited, whereas in practice it is likely that there is a certain maximum, as postulated by van Bunnik et al. (2014) that assumed the force of infection to be limited by a maximum exposure capacity for recipient animals. However, in our study models including a maximum exposure capacity rendered a model that did not converge, which might indicate that the maximum exposure capacity was not yet reached at the end of the experiment.

In poultry practice, the interventions studied in this experiment could be used to control the spread of ESBL-producing *E. coli*. CE product could be supplied on the farm via the drinking water system. Supply should be done as soon as possible after hatching, before exposure to ESBL-producing *E. coli* occurs. In this study compartmentalization, including separation of feed- and water systems and strict hygiene measures, reduced transmission of ESBL-producing *E. coli* but could not prevent it. In practice, with less strict hygiene measures, the effects might be smaller.

## Conclusion

Overall, our study shows that CE is a useful intervention tool to prevent colonization of ESBL-producing bacteria after challenge with a low dose in the first week in a broiler flock. Transmission within a flock could be delayed by compartmentalization, however as soon as ESBL-producing bacteria are excreted and accumulate in the environment spread to other birds seems inevitable. Therefore, compartmentalization of large flocks into smaller groups of birds, which is for instance more common in breeding flocks at higher levels of the broiler production chain, could be combined to enhance the efficacy of other interventions. CE products could be supplied to young chicks after hatching at different levels of the broiler production pyramid to prevent colonization of birds. The insights provided by this study may provide a basis for further developments toward practically applicable measures to further reduce antimicrobial resistance in poultry.

## Data Availability Statement

Raw sequence data were deposited into the Sequence Read Archive (SRA) at the NCBI, under accession number PRJNA647260.

## Ethics Statement

The animal study was reviewed and approved by Dutch Central Authority for Scientific Procedures on Animals and the Animal Experiments Committee of Utrecht University (Utrecht, Netherlands) under registration number AVD108002015314.

## Author Contributions

AD, EF, JvdG, FV, DC, DM, and AS: experiment design. AD and FV: experiment execution. AD, JK, and EF: data analysis. AD and JK: manuscript writing. All authors discussed, read, contributed to, and approved the final manuscript.

## Disclaimer

The views expressed are purely those of the author(s) and may not in any circumstances be regarded as stating an official position of the European Commission.

## Conflict of Interest

DC is currently employed by the Research Executive Agency. The remaining authors declare that the research was conducted in the absence of any commercial or financial relationships that could be construed as a potential conflict of interest.
